# Antistatic Fibers for High-Visibility Workwear: Challenges of Melt-Spinning Industrial Fibers

**DOI:** 10.3390/ma13112645

**Published:** 2020-06-10

**Authors:** Rudolf Hufenus, Ali Gooneie, Tutu Sebastian, Pietro Simonetti, Andreas Geiger, Dambarudhar Parida, Klaus Bender, Gunther Schäch, Frank Clemens

**Affiliations:** 1 Laboratory for Advanced Fibers, Empa, Swiss Federal Laboratories for Materials Science and Technology, Lerchenfeldstrasse 5, 9014 St. Gallen, Switzerland; ali.gooneie@empa.ch (A.G.); pietro.simonetti@outlook.com (P.S.); dambarudhar.parida@empa.ch (D.P.); 2 Laboratory for High Performance Ceramics, Empa, Swiss Federal Laboratories for Materials Science and Technology, Überlandstrasse 129, 8600 Dübendorf, Switzerland; tutu.sebastian@empa.ch (T.S.); geigerandreas@web.de (A.G.); frank.clemens@empa.ch (F.C.); 3 EMS-CHEMIE AG, Business Unit EMS-GRILTECH, Via Innovativa 1, 7013 Domat/Ems, Switzerland; klaus.bender@emsgriltech.com (K.B.); gunther.schaech@emsgriltech.com (G.S.)

**Keywords:** bicomponent melt-spinning, safety workwear, nanocomposite, antistatics, carbon black

## Abstract

Safety workwear often requires antistatic protection to prevent the build-up of static electricity and sparks, which can be extremely dangerous in a working environment. In order to make synthetic antistatic fibers, electrically conducting materials such as carbon black are added to the fiber-forming polymer. This leads to unwanted dark colors in the respective melt-spun fibers. To attenuate the undesired dark color, we looked into various possibilities including the embedding of the conductive element inside a dull side-by-side bicomponent fiber. The bicomponent approach, with an antistatic compound as a minor element, also helped in preventing the severe loss of tenacity often caused by a high additive loading. We could melt-spin a bicomponent fiber with a specific resistance as low as 0.1 Ωm and apply it in a fabric that fulfills the requirements regarding the antistatic properties, luminance and flame retardancy of safety workwear.

## 1. Introduction

Antistatic properties in safety workwear are required in order to reduce its electrical resistivity. If friction-induced charge cannot dissipate across the fabric surface and discharge, it may build up and create sparks and static electricity, which can be highly dangerous. Therefore, antistatic clothing is required to prevent fire and explosions while working with flammable liquids and gases, and to avert damage to sensitive electrical components. Antistatic properties can be achieved by a surface treatment that absorbs moisture, resulting in a thin conductive film on the fabric [1,2]. The commercial fiber Resistat^®^ [3], a polyamide 6 (PA6) fiber with a carbon black (CB) coating, is one such example. In respective garments, the treatment can fade or rub off, and the resistivity is influenced by the atmospheric humidity and can increase in dry environments.

In order to make synthetic fibers intrinsically antistatic or conductive, fillers such as CB, carbon nanotubes (CNTs), graphene or metal powders are often used as electrically conductive additives [1,4,5]. Typically, the mechanical properties of synthetic fibers change significantly when conductive fillers are used. Since fibers with fillers have very low tenacity, bicomponent fibers with an antistatic compound as a minor element are an interesting approach [6,7]. Respective bicomponent fibers are already commercially available, e.g., “Belltron” (side-by-side) by Kanebo [8] or “Antistat” (core-sheath) by Perlon [9]. In this study, we take Belltron B31 [8] as the reference, with the fiber having an electrical resistivity of ~66 Ωm, and the conductive part of the bicomponent fiber one of ~6 Ωm. In this study, these resistivities are applied for comparison with bicomponent fibers or antistatic compounds, respectively.

Another problem with carbon-based fillers is that they lead to unwanted dark colors in the resulting fibers. This reduces the visibility of the corresponding luminous fabrics used for high-visibility (hi-viz) workwear. Garments with hi-viz features are meant to protect workers exposed to risks from vehicles and heavy equipment. Higher visibility can be achieved by using a bicomponent fiber approach, when only a small part of the conductive phase is connected to the surface of the fiber, thus reducing the appearance of the black color. The goal of our study was to achieve good electrical conductivity and simultaneously attenuate the undesired dark color. To enable stable fiber melt-spinning, good electrical conductivity should be realized with low carbon content.

In general, the electrical resistivity of a polymeric material decreases with an increasing content of a conductive filler like carbon black [10]. Gulrez et al. give a good overview of different conductive fillers (e.g., CB, CNTs, graphite, graphene and metal nanoparticles) in polyolefin-based thermoplastics [11]. The formation of the conductive pathways of the fillers is described by the percolation curve, which illustrates the relationship between the quantity of added CB and the achieved electrical resistivity [12]. As soon as the particles start to form an interconnected network, i.e., percolation, the electrical resistivity drops dramatically.

The shape of the percolation curve is mainly affected by the state of dispersion, morphology, content, and intrinsic properties of the conductive filler [10]. It is also well known that the polymer–polymer, polymer–filler and filler–filler interactions play an important role in the equilibrium microstructure of the (nano)composite and hence control the achieved electrical conductivity [13]. Theoretical and experimental investigations suggest that mixing carbon nanoparticles with different shapes and aspect ratios can lead to developing more continuous networks and thus increase the electrical conductivity at a fixed carbon content [10,13,14,15,16]. Molecular simulations have shown that by increasing the aspect ratio, the equilibrium microstructure of nanoparticles in a polymer matrix changes from a random dispersion to a self-assembled morphology and eventually to a bridging self-assembled network [14]. Besides the particle shape, a range of electrical conductivity can be achieved by varying the amount of filler added to the polymer. In case of CNTs and carbon nanofibers (CNFs), enhanced polypropylene (PP) with a high electrical conductivity can be achieved at relatively low filler loading [11]. Moreover, mechanical models have been developed to address the overall behavior in such polymer (nano)composites [17]. Based on this knowledge, researchers have already investigated the combination of CNT and CB fillers in polymers [18].

In this study, a bicomponent melt-spinning technique to grant antistatic properties to synthetic fibers is reported. First, different conventional approaches to achieve sufficient electrical conductivity with the lowest possible carbon content are assessed, scrutinizing combinations of CBs and CNTs in polyamide, as well as highly loaded CB compounds blended with an immiscible neat polymer (double-percolated conductive network) [19,20,21]. Secondly, elaborate melt-spinning trials are presented, performed on a custom-made pilot line to determine what material and cross-sectional combinations result in fibers with low electrical resistivity, attenuated blackness and sufficient tensile properties. Finally, the bicomponent antistatic fiber concept is transferred to industrial-scale production and followed through to a prototype antistatic hi-viz safety jacket.

## 2. Materials and Methods

### 2.1. Polymers and Compounds

The neat polymers used to dilute antistatic masterbatches by compounding were the low-densitiy polyethylene (LDPE) 1700 MN 18 C, the medium-density polyethylene (MDPE) 1020 FE 30, the high-density polyethylene (HDPE) 2055 MN, the polyamide 6 (PA6) Grilon A26, and the polyamide 12 (PA12) Grilamid L 20. The base polymer used for fiber melt-spinning was the PA6 Grilon F 34 NL. The commercial antistatic compounds included in this study were the CB-based pigment masterbatches Grilamid L 20 EC, Palamid Black 00-6405 and the polyethylene (PE) compound ColColor RKK E 40/FP, as well as the CNT-containing masterbatch PlastiCyl PA1502. Grilamid FE 11384 is an in-house antistatic compound developed by EMS-CHEMIE (Domat/Ems, Switzerland); compounds No. 6081 and 6082 are 50:50 and 85:15 mixtures of Grilamid L 20 EC and PlastiCyl PA1502, respectively, while compound No. 6083 is a 90:10 mixture of Grilamid FE 11384 and PlastiCyl PA1502. Compounds No. 6237 and 6739 are 50:50 mixtures of ColColor RKK E 40/FP with Grilon F 34 NL and Sabic LDPE, respectively, prepared on a co-rotating 36 L/D twin-screw extruder (Collin, Maitenbeth, Germany) by Empa. The pigment masterbatch Palamid White 00-2305 was used to attenuate the dark color of the carbon. Details of the compounds are given in Table 1.

### 2.2. Extrusion and Melt-Spinning

**Figure 1:**
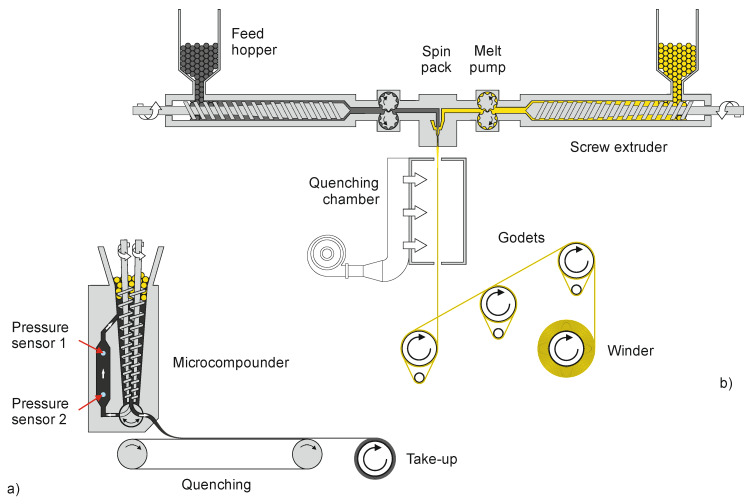
Schematic drawing of (**a**) the micro compounder with a backflow channel and quenching and take-up units, and (**b**) of the pilot melt-spinning plant (diameters of godets and winder spool are 100 mm). By way of illustration, the colorless polymer is represented in yellow.

The polymer compounds were homogeneously mixed using the microcompounder Minilab (Thermo Fisher Scientific, Karlsruhe, Germany) to produce undrawn filaments with high carbon filler content (Figure 1a). Here, the mixture was fed with a top-feeder into a co-rotating conical twin-screw extruder at a speed of 100 rpm and mixed at a temperature of 250 °C. The mixture was cycled towards a backflow slit die channel, equipped with two pressure sensors. After reaching the equilibrium in the pressure sensors, the valve was switched towards a die with diameter 0.5 mm. The filaments were extruded onto a conveyor belt and collected.

For the experiments, masterbatches No. 6107 and 6111, both with 40 wt% CB, were diluted with the thermoplastics listed in Table 1, to investigate the effect of the CB content on the resistivity. To investigate the effect of additional CNTs, CB masterbatch No. 6107 and CNT masterbatch No. 6065 were diluted with PA12 (No. 5793). Details of the respective compounds and the resulting extrudates are given in Table S1 of Supplementary Materials.

**Figure 2:**
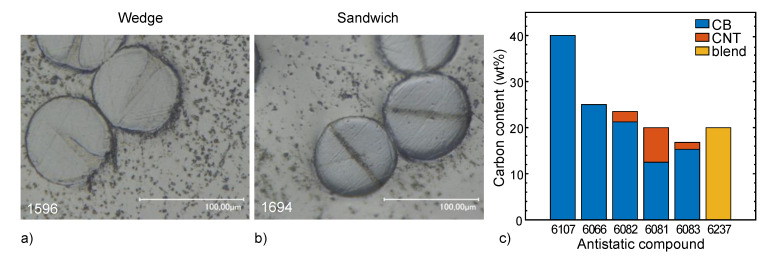
Illustration of the antistatic compounds: optical microscopy images of the two types of fiber cross-sections produced: (**a**) wedge, and (**b**) sandwich. (**c**) Carbon content and type of filler in melt-spun bicomponent fibers.

The bicomponent fibers were melt-spun in a custom-made pilot plant (Figure 1b), originally built by Fourné Polymertechnik (Alfter, Germany), and described in detail elsewhere [22]. Two types of spinnerets were applied, yielding either “wedge” (Figure 2a) or “sandwich” (Figure 2b) fiber cross-sections. The major component of the bicomponent fiber consisted of PA6 (polymer No. 5528) with 3 wt% and 7 wt% of white masterbatch (MB No. 6183), resulting in 0.9% and 2.1% TiO_2_ content in the major fiber part, respectively. Six different antistatic components were introduced (13 or 20 vol%) as minor components (Figure 2c): PA6 with 40 wt% CB (No. 6107), PA12 with 25 wt% CB (No. 6066), PA12 with 21.25 wt% CB and 2.25 wt% CNT (No. 6082), PA12 with 12.5 wt% CB and 7.5 wt% CNT (No. 6081), PA12 with 15.3 wt% CB and 1.5 wt% CNT (No. 6083), and a 50:50 blend of PE containing 40 wt% CB with PA6 (No. 6237).

The two polymer composites were fed from two separate single-screw extruders (Fourné Polymertechnik, Alfter, Germany; length to diameter ratio of 25, major component: 18 mm diameter, minor component: 14 mm diameter). Metering pumps (Mahr, Göttingen, Germany) with nominal throughputs of 5.4 and 0.8 cm^3^/min (bicomponent ratio, 87:13), as well as 4.8 and 1.2 cm^3^/min (bicomponent ratio 80:20), transferred the melts into the spin pack. The respective spin pressures and processing temperatures can be found in Table S2. The bicomponent fiber exited the spinneret into the quenching chamber, where it was cooled by air in order to solidify before drawing. Finally, the fiber was taken up, drawn by three heated godets (100 mm diameter, decreasing temperatures: 85, 50 and 30 °C), and spooled onto a bobbin (100 mm diameter). The take-up velocity was 300 m/min, and the draw ratio, namely the ratio between the winder speed and that of the take-up godet, was varied within the range 1–4 (resulting in respective winding speeds of 300–1200 m/min).

### 2.3. Physical Properties

The resistivity of the filaments produced by the microcompounder, i.e., their length and cross-section specific electrical resistance, was evaluated with a four-point probes method that uses separate pairs of current-carrying and voltage-sensing electrodes to make more accurate measurements when the resistance is low. The distance between the four probes, connected to a multimeter (SourceMeter 2450, Keithley Instruments, Cleveland, OH, USA), was 10 mm. A two-point measurement was applied to assess the resistivity of the melt-spun bicomponent fibers, as their electrical resistance was high enough in all cases. Here, the fibers were fixed with a conductive silver paste (Acheson, Agar Scientific, Stansted Essex, UK) on two probes with a distance of 10 mm, connected to the multimeter.

The tensile characteristics of the melt-spun fibers were determined using a Statimat ME+ (Textechno Herbert Stein, Mönchengladbach, Germany) with a 10 N load cell. A series of 10 specimens were tested for each fiber type using a gauge length of 100 mm and a cross head speed of 200 mm/min. The fineness was calculated from the weight of a 100 m long fiber sample.

To study the fiber cross-section, fibers were embedded in epoxy resin and cross-sections prepared with the polisher EcoMet 250 Pro (Buehler, Esslingen am Neckar, Germany). Optical microscopy pictures were taken using a VHX-1000 (Keyence, Mecheln, Belgium) system. Scanning electron microscopy (SEM) was conducted on a Hitachi S-4800 SEM (Hitachi High-Technologies, Krefeld, Germany). As prepared, the fiber samples were coated with Au/Pd (5 nm) prior to analysis.

The surface color of a fiber bobbin was measured using the dual-beam spectrophotometer Datacolor 550 (Datacolor, Rotkreuz, Switzerland) fitted for reflectance measurements, in a wavelength range between 360 nm and 700 nm. The bobbin was attached to an integrating sphere (diameter 152 mm) and illuminated through a slit of defined size, in order to detect the spectrum of the reflected light.

## 3. Results and Discussion

### 3.1. Assessment of Different Approaches to Reduce the Resistivity of Compounds

**Figure 3:**
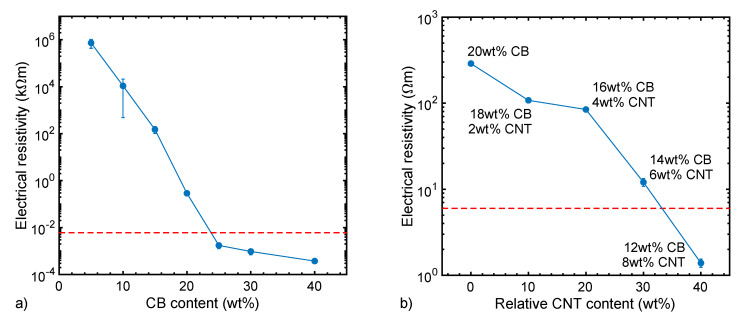
Electrical resistivity of (**a**) polyamide (PA) filaments produced by microcompounding, as a function of CB content; and (**b**) compounded/extruded filament as a function of relative CNT addition with respect to the total carbon content (i.e., CB + CNT), which is kept at 20 wt%. For clarification, the absolute CB and CNT concentrations of the respective compounds are stated in the graph. The error bars represent double the standard deviation (some of the error bars are too small to be visible; see Table S1). For comparison, the resistivity of the conductive part of the commercial bicomponent fiber Belltron B31 is plotted as dashed line.

Before discussing the properties of melt-spun polymer fibers, we establish an assessment of potential approaches to produce conductive compounds for later use in fibers. The electrical conductivity of polymers with carbon fillers is first and foremost influenced by the filler content. Upon diluting the PA6 compound containing 40 wt% CB (No. 6107) with neat PA12 (No. 5793), the electrical resistivity increases (Figure 3a). Figure 3a shows that, by adding CB in steps of 5 wt%, the resistivity of PA gets roughly 100 times lower per step, until the percolation threshold is reached and the resistivity levels out. Without drawing the filament, a good enough resistivity, compared to the conductive part of the commercial fiber Belltron B31 (~6 Ωm), is reached at about 24 wt% CB. Follow-up fiber spinning experiments revealed that a filler content above 20 wt% resulted in the clogging of the filters and spinneret over time.

**Figure 4:**
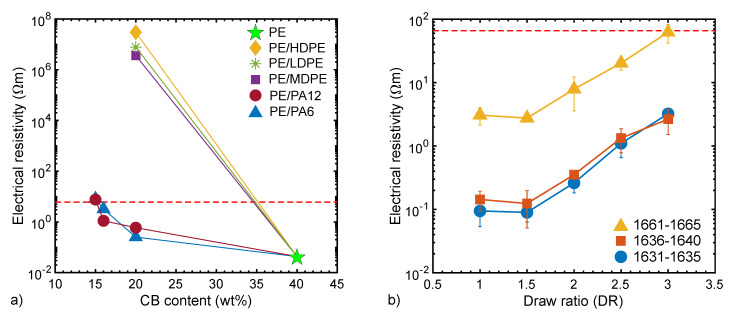
(**a**) Electrical resistivity of compounded/extruded filament of PE masterbatch No. 6111, blended with different polymers. PE = No. 6111; PE/PE: 50% No. 6111 diluted in HDPE, LDPE and MDPE, respectively; PE/PA12: 37.5%, 40% and 50% No. 6111 diluted in No. 5793; PE/PA6: 37.5%, 40% and 50% No. 6111 diluted in No. 5432. The miscible compounds show a very high resistivity, whereas the immiscible blends reveal a resistivity in the range of the value of the conductive part of the commercial bicomponent fiber Belltron B31 (~6 Ωm, dashed line). (**b**) Electrical resistivity of selected melt-spun bicomponent fibers with 13 vol% antistatic compound (Table S3) as a function of draw ratio (DR). For comparison, the resistivity of the commercial bicomponent fiber Belltron B31 (~66 Ωm) is plotted as dashed line. The error bars represent double the standard deviation (see Tables S1 and S3); some of the error bars are too small to be visible.

Upon diluting a PE compound containing 40 wt% CB (No. 6111) with PE (No. 887) in order to achieve 20 wt% CB in the mixture, the resistivity reaches 0.23 ± 0.01 Ωm after once mixing in the twin-screw extruder Collin (Table S1). Upon mixing the same compound (No. 6739) a second time in the same way, the resistivity increases a thousand-fold to 0.32 ± 0.15 kΩm (Table S1), which can be explained by the breakdown of percolated networks of aggregated CB. Compounding a similar material combination (50 wt% LDPE with 50 wt% 6111) in the microcompounder leads to an abrupt increase in resistivity up to 7.8 ± 0.4 MΩm (Table S1, Figure 4a). This trend shows the significant effect of intensive mixing on the formation and breakdown of percolated networks of conductive CB fillers in the polymer matrix, as explained by the so-called “aggregate-network” model [23].

Fillers’ properties such as their particle size and aspect ratio are known to influence the electrical conductivity of polymer (nano)composites [14,15,16]. In particular, the dispersion quality, aspect ratios of carbon nanoparticles, and formation of nanoparticle networks with continuous pathways for efficient electron transport are largely interconnected. Various studies have shown the synergistic effect of a CB–CNT hybrid filler on the electrical conductivity of the polymer composite [24]. Thus, we evaluated different CB–CNT combinations between 5 and 40 wt% carbon, with 0–100 wt% CNT content. Due to the higher area to weight ratio of CNTs, a lower filler loading is needed to reach the percolation threshold, which results in a reduced resistivity (Figure 3b). Figure 3b shows that, with high enough CNT loading, the overall carbon content to reach a good enough resistivity can be below 20 wt%.

Polymer nanocomposites based on CB–CNT mixtures still retain their undesired black color despite their advantage of higher conductivity at lower carbon contents. In an attempt to further reduce the blackness of the extrudates while preserving the electrical conductivity, the PA6 and PE compounds containing 40 wt% CB (No. 6107 and 6111, respectively) were diluted with either neat polyethylenes (HDPE, MDPE or LDPE) or polyamides (PA6 and PA 12, No. 5432 and 5793, respectively). As a result, carbon particles mainly accumulated in one phase or at the interface of the polymers and thus formed the so-called double-percolated conductive networks [20,25,26]. The concept of double-percolated polymer blends seems to work as long as the polymers are immiscible (Figure 4a). Mamunya [27] suggested that, among immiscible polymers, CB tends to accumulate more in the polymer with lower surface tension. The distribution of CB in two different polymer phases can be predicted based on their interfacial tension and wetting behavior [26]. The distribution tendency of CB in immiscible polymer blends found in literature is summarized in Table 2 (see also [28]). Based on the surface energies of PE [29], PA6 [29,30], PA12 [30,31] and CB [21], as well as their temperature dependencies, we estimated the wetting parameters to determine the distribution of CB in our blends [31]; see Table 2. We predict that CB selectively locates either at the interface of PE/PA6 or in the PE phase of PE/PA12 blends. Thus, a double-percolated conductive network can be envisioned in both immiscible blends. In consequence, high conductivity could be achieved at lower carbon contents. However, the transfer of this concept to bicomponent antistatic fibers failed, as is explained in the next section.

### 3.2. Physical Properties of Bicomponent Fibers

Based on the results presented so far, it appears that the most convenient approach to overcome the intrinsic resistivity of synthetic fibers is to add carbon particles in high concentrations to the polymer matrix and melt-spin them into bicomponent fibers. In bicomponent melt-spinning, two polymers of different chemical/physical natures are extruded from one spinneret to form a single fiber [6]. This approach, with an antistatic compound as a minor element, prevents a severe loss of the tensile strength of the melt-spun fibers caused by a high additive loading.

A design of experiment (DoE) was chosen that covers a theoretical maximum of 336 combinations. In the end, 110 different bicomponent fibers were melt-spun and analyzed (Table S3). The sandwich cross-sections were regular in shape for all respective fibers (Figure 2b or Figure S1a–c). The shape of the wedge cross-sections, on the other hand, varied strongly as a function of the viscosity differences of the two compounds constituting the bicomponent fibers (Figure 2a or Figure S1d–i). For all cross-sections, the shape was maintained when the fibers were drawn (Figure S1a–f).

**Figure 5:**
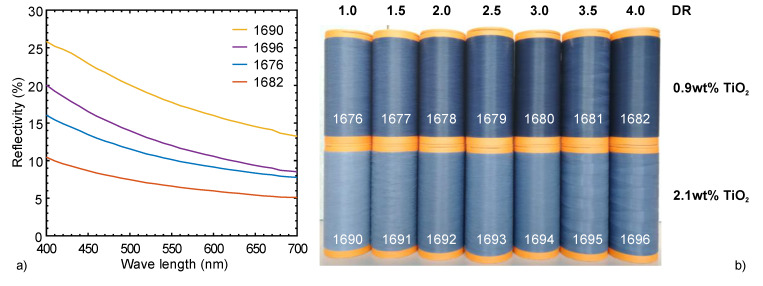
Grayness of selected filaments as a function of the draw ratio (DR) and TiO_2_ content: (**a**) reflectivity (in %) measured directly on the bobbins; (**b**) photograph of fiber bobbins.

TiO_2_ was used to partially bury the conductive element inside a dull bicomponent fiber, and to attenuate its undesired dark color. As an effect of increased draw ratio, we noticed that the scattering efficiency of the TiO_2_ pigments decreased with increasing spatial dispersion, while the coverage of the carbon black was largely unaffected (Figure 5).

**Figure 6:**
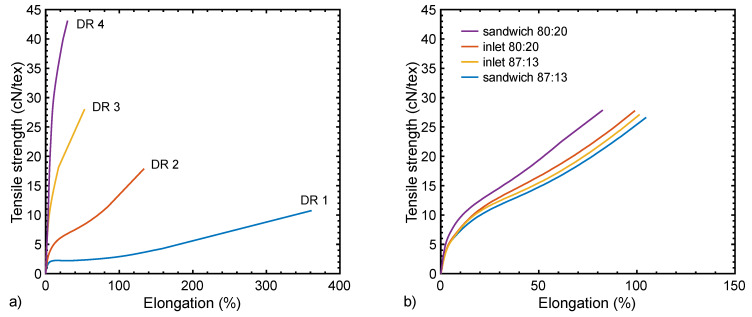
Average tensile stress–strain curves of selected fibers containing 13% of PA12 with 15.3 CB and 1.5 CNT as the antistatic component: (**a**) sandwich cross-section fiber at different draw ratios (1: 1647, 2: 1649, 3: 1651, 4: 1653); (**b**) wedge (80/20: 1623, 87/13: 1628) and sandwich (80/20: 1644, 87/13: 1650) cross-section fibers with draw ratio 2.5.

The tensile properties of selected fibers are listed in Table S4. Additionally, Figure 6a shows the load–strain behavior of melt-spun bicomponent fibers, which is typical for PA6 fibers [42,43]. In the undrawn state (DR 1), the fibers show considerable plastic deformation under stress. With an increasing draw ratio, the fibers gain in stiffness and resilience. A draw ratio of 4 is about the maximum that can be achieved without breaking the fiber, which corresponds to an elongation of 300% of the undrawn (DR 1) fiber. These findings indicate that the antistatic compound has an industrially tolerable impact on the tensile properties, as long as its proportion is small (at or below 20 vol%). As expected, the specific tensile strength (in cN/tex) is reduced in comparison to a pristine PA6 filament (Table S4), mainly since the antistatic compound contributes to the weight but not to the tensile strength. Tex, a direct measure of the linear fiber density, is the mass in grams per 1000 m of the fiber.

Figure 6b also illustrates that the influence of the cross-section type on the load–strain behavior is small. The maximum tensile strength seems to be slightly higher with the wedge cross-section, most probably due to a smaller influence on the integrity of the fiber (see Figure 2a). Furthermore, the increased stiffness of the sandwich fiber with a higher antistatic portion can be explained by a mobility constraint of the load-bearing part by the antistatic fraction. Such a constraint seems not to affect the wedge fibers, most probably because of the above-mentioned smaller influence on the load–strain behavior.

**Figure 7:**
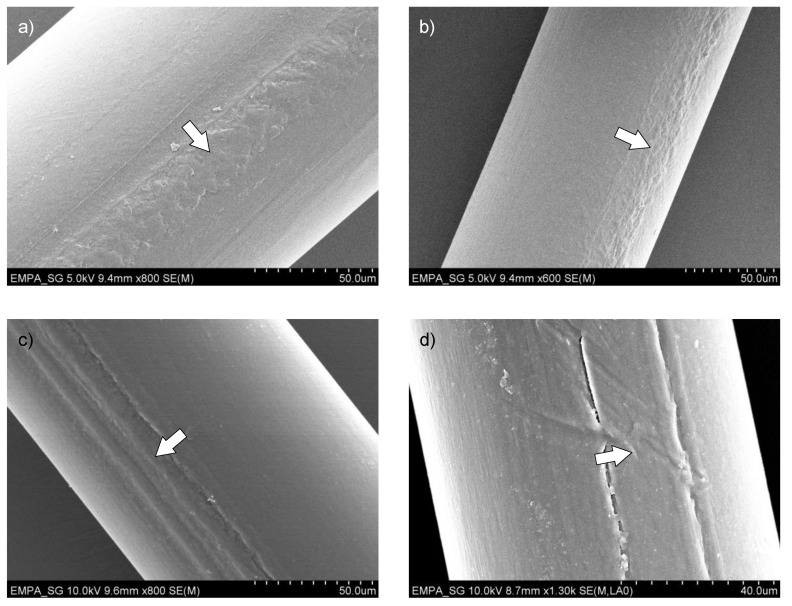
SEM images of selected fiber surfaces. (**a**,**b**) Sandwich fibers with antistatic compound No. 6066 (25% CB in PA12), draw ratios 1 (No. 1581, (**a**)) and 2 (No. 1583, (**b**)). (**c**,**d**) Sandwich fibers with antistatic compound No. 6237 (50:50 mixture of PA6 and PE with 40% CB, draw ratios 1 (No. 1669, (**c**)) and 2 (No. 1671, (**d**)). Arrows indicate the antistatic compound.

Scanning electron microscopy pictures of selected sandwich fiber surfaces (Figure 7) show the following. The surface of the antistatic compound shows a higher roughness than that of pure PA6, which can be explained by the tendency of CB to agglomerate within the polymer matrix. When the base polymer of the antistatic compound and the main fiber component are miscible (Figure 7a,b), the sandwich filling is perfectly integrated in the fiber, but if they are immiscible (Figure 7c,d), the two components tend to separate, which could lead to fibrillation when a mechanical stress is applied. However, no transversal cracks were found in the sandwich fillings of the drawn fibers (Figure 7b,d), thus the increasing electrical resistivity at higher draw ratios (Figure 4b) cannot be explained by a rupture of the antistatic compound.

The increase in the resistivity when the fiber is melt-drawn can be ascribed to a spatial dilution of the carbon filler (Figure 4b). Here, a draw ratio (DR) of 3 still gives low enough resistivity values. As this is also a reasonable DR for PA6, we mainly consider this DR for the following analysis. As long as the carbon content is above a certain value, the resistivity is only affected by the conductivity of the carbon composite and not by its content ratio within the bicomponent fiber, as shown in Figure 4b. In other words, it is sufficient that only a small portion of the bicomponent fiber is conductive.

**Figure 8:**
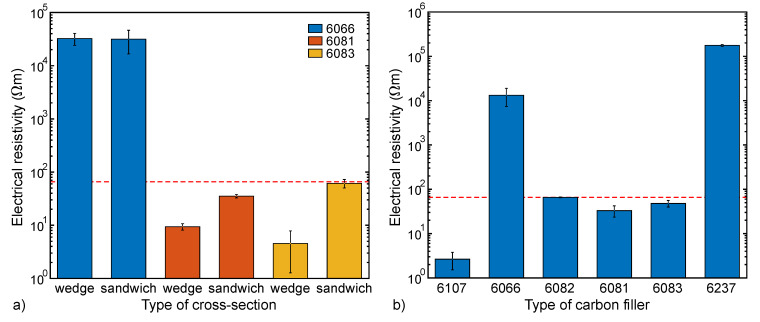
Resistivity of melt-spun bicomponent fibers (DR 3) as a function of the (**a**) fiber cross-section (fibers with 20 vol% antistatic compound) and (**b**) type of carbon filler (sandwich fibers with 13 vol% antistatic compound). For comparison, the resistivity of the commercial bicomponent fiber Belltron B31 (~66 Ωm) is plotted as a dashed line. The error bars represent double the standard deviation (some of the error bars are too small to be visible; see Table S3).

At the same bicomponent ratio (20:80), the shape of the cross-section has no influence when only CB is used (Figure 8a). However, when CNTs are added, the resistivity is reduced in case of the wedge structure. This is probably due to the spinneret channels, which are comparatively narrower to achieve the wedge cross-section. The resulting higher shear improves the dispersion state of the CNTs by breaking the CNT aggregates, leading to lower resistivity [44,45].

Figure 8b shows the influence of the type of filler on the resistivity. With 40 wt% CB (6107), even with a DR of 3, the percolation threshold is far exceeded and the resulting resistivity is 25 times lower than that of the commercial fiber. With 25 wt% CB (6066), the dilution of the carbon filler at a draw factor of 3 increased the resistivity by a factor of ~1000, whereas the twin-screw extruded undrawn filaments had previously shown sufficient conductivity (Figure 3a). The combination of CB and CNTs, on the other hand, resulted in good conductivity (i.e., low enough resistivity) at an overall carbon content below 25 wt% (6081–6083). Considering the approach with a 50:50 polymer–polymer blend with carbon black in only one of the immiscible polymers (6237), the conductivity was lost after the melt-spinning the blend. We believe that this is due to breakup of the conductive domains within the nonconductive polymer in the narrow channels of the spinneret, which led to losing the double-percolation.

In conclusion, we found that the carbon content can be reduced by applying a mixture of CB and CNTs in the antistatic compound, and by improving the dispersion state of CNTs in the narrow melt-flow channels of a bicomponent spinneret [45]. On the other hand, the grayness can be attenuated by a combination of a low carbon composite ratio in the bicomponent fiber and a high TiO_2_ loading in the major polymer component. Such an approach would also guarantee good mechanical behavior of the melt-spun fibers.

### 3.3. Prototype Production of Antistatic Hi-Viz Workwear

Upscaling trials on an industrial-scale spinning plant, using the sandwich cross-section approach, showed that all CNT-containing composites led to severe spinning instabilities, finally resulting in filter and spinneret blockage. The main reason for this setback is that the industrial-scale spin-packs and spinnerets are not typically optimized for such a kind of materials. Since developing a new design for the processing equipment was beyond the scope of this research, we were forced to resort to our CNT-free material for industrial melt-spinning. In consequence, the final upscaling trials were performed with the 40 wt% CB compound (No. 6107), the only feasible conductive composite left in this study.

For both the antistatic composite and main bicomponent fiber polymer (No. 5528), PA6 was used for the upscaling melt-spinning trials at EMS-CHEMIE AG (Domat/Ems, Switzerland). Due to co-extrusion viscosity mismatch, adding TiO_2_ to the base polymer also led to spinning instabilities; in consequence, the final upscaling trials were performed without TiO_2_. The lowest bicomponent ratio achievable with the available industrial-scale melt-spinning plant over a prolonged time was 43 vol% of antistatic compound. Although the processing conditions were far from optimal, we still proceeded to the production of the respective antistatic staple fibers (fineness, 1.2 ± 0.3 tex; nominal length, 51 mm). The resulting fibers reached a resistivity of less than 0.1 Ωm, which is three orders of magnitudes better than that of the commercial fiber Belltron B 31 (~66 Ωm; nominal fineness, 0.3 tex) that we took into consideration as a reference. The overall carbon black content in the fiber, calculated from the CB content of the masterbatch and the bicomponent ratio of the fiber, was ~17 wt%.

**Figure 9:**
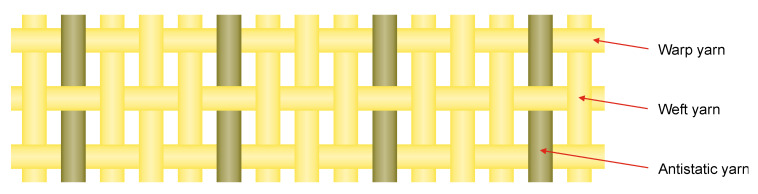
Plain weave fabric with every fourth of the weft yarns being antistatic, the rest being neat flame-retardant staple fiber yarns.

In the next step, the Rieter Spin Center (Winterthur, Switzerland) produced a staple fiber yarn by ring spinning, having a fineness of 40 tex. The yarn was composed of 95 wt% flame retardant polyester staple fibers (Trevira CS, fineness 0.33 tex, nominal length 60 mm) and only 5 wt% of above antistatic staple fiber, resulting in a CB content of ~0.9 wt% concerning the yarn. With this antistatic yarn, Jenny Fabrics AG (Niederurnen, Switzerland) produced a plain weave fabric (Figure 9), with only one out of eight yarns being antistatic (none of the warp yarns, only every fourth of the weft yarns), the rest being neat flame-retardant staple fiber yarn (Trevira CS, fineness 37 tex). Thus, the final CB content of the fabric was ~0.1 wt%. Finally, the fabric was finished with a luminescent dye by AG Cilander (Herisau, Switzerland). With this fabric, a hi-viz safety workwear jacket was produced by Hüsler Berufskleider AG (Sirnach, Switzerland) and positively tested regarding the most relevant requirements, i.e., surface resistivity (EN 1149-5 [46]), luminescence (EN 471 [47]) and flame retardancy (UL94 [48]).

## 4. Conclusions

The use of antistatic fibers in safety workwear is an established technology to prevent friction-induced sparks that can harm delicate equipment or provoke fire and explosions in combustible environments. Despite their blackness, especially objectionable in hi-viz applications, and its negative influence on the tensile properties of fibers, carbon (nano)particles (CB and CNTs) are still the most promising candidates for antistatic compounds. In this study, we assess the potential of various conventional methods of implementing such particles in polymers, in order to increase the electrical conductivity of the host. A rather comprehensive overview of these approaches is offered, which reveals the challenges of upscaling such materials from the lab to pilot to the industrial production of melt-spun fibers.

We show that the embedding of a conductive compound into a bicomponent melt-spun fiber, as a minor component within a TiO_2_-pigmented main polymer, can result in a fiber with reduced grayness and still-sufficient conductivity and tensile strength. It is discussed that the combination of CB and CNT within one polymer, as well as the blending of a CB compound with a pure immiscible polymer, can both lead to reduced electrical resistivity at maintained carbon content. However, the attempt to melt-spin respective antistatic compounds in a bicomponent approach resulted either in severe processing instabilities or in total failure of the anticipated electrical performance.

Nevertheless, we could successfully develop and produce an antistatic fiber at the industrial scale with an electrical resistivity below 0.1 Ωm, which is about a thousand times better than that of the existing commercial fibers. Out of these fibers, we produced a staple fiber yarn and finally a woven fabric, which was finished with a luminescent dye. From this fabric, a safety workwear jacket was tailored, which fulfilled the relevant standards regarding resistivity, luminescence and flame retardancy.

We conclude by emphasizing that there is still a lot of space for improvement. To realize accordingly optimized antistatic fibers on a large scale, for instance, an auxiliary extruder with comparably small throughput would be required, as well as a special spinneret that takes the viscosities of the carbon composite and the TiO_2_-pigmented main polymer into account.

## Figures and Tables

**Table 1:** Polymers and compounds used in this study. Carbon black (CB), carbon nanotube (CNT) and TiO_2_ content are specified by the provider.

Code/No.	Product Name	Provider	Base Polymer	CB Content (wt%)	CNT Content (wt%)	TiO_2_ Content (wt%)
LDPE	LDPE 1700 MN 18 C	Total	LDPE	-	-	-
MDPE	MDPE 1020 FE 30	Arkema	MDPE	-	-	-
HDPE	HDPE 2055 MN	Total	HDPE	-	-	-
887	Sabic LDPE	Sabic	LDPE	-	-	-
5432	Grilon A26	EMS-CHEMIE	PA6	-	-	-
5528	Grilon F 34 NL	EMS-CHEMIE	PA6	-	-	-
5793	Grilamid L 20	EMS-CHEMIE	PA12	-	-	-
6065	PlastiCyl PA1502	Nanocyl	PA12	-	15	-
6066	Grilamid L 20 EC	EMS-CHEMIE	PA12	25	-	-
6067	Grilamid FE 11384	EMS-CHEMIE	PA12	17	-	-
6081	L-2597 KTI-2	EMS-CHEMIE	PA12	12.5	7.5	-
6082	L-2598 KTI-3	EMS-CHEMIE	PA12	21.25	2.25	-
6083	L-2599 KTI-5	EMS-CHEMIE	PA12	15.3	1.5	-
6107	Palamid Black 00-6405	BASF	PA6	40	-	-
6111	ColColor RKK E 40/FP	Evonik	PE	40	-	-
6183	Palamid White 00-2305	BASF	PA6	-	-	30
6237	-	-	PE/PA6	20	-	-
6739	-	-	PE/PE	20	-	-

**Table 2:** Distribution tendency of CB in immiscible polymer blends. Polymers considered: polymethyl methacrylate (PMMA), polypropylene (PP), polyoxymethylene (POM), polyvinylidene fluoride (PVDF), ethylene vinyl acetate (EVA), polystyrene (PS) and acrylonitrile butadiene styrene (ABS).

Blend Composition			Distribution Tendency of CB	Reference
PMMA	PP	CB	Interface	Sumita et al. [20]
PMMA	HDPE	CB	Interface	Sumita et al. [20]
LDPE	PP	CB	LDPE	Mamunya [27]
LDPE	POM	CB	Interface	Mamunya [27]
HDPE	PVDF	CB	HDPE	Feng et al. [32]
PA66	PP	CB	PA66	Zoldan et al. [33]
PA6/6-9	PP	CB	PA6/6-9	Tchoudakov et al. [34]
PP	EVA	CB	EVA	Huang et al. [35]
PP	PS	CB	PS	Tan et al. [36]
LDPE	EVA	CB	LDPE	Yu et al. [37]
PS	PMMA	CB	PS	Pan et al. [38]
HDPE	PP	CB	HDPE	Xu et al. [39]
PP	PA6	CB	PA6	Chen et al. [40]
ABS	PA6	CB	PA6	Wu et al. [41]
PS	PA6	CB	PA6	Xu et al. [39]
PMMA	PA6	CB	PA6	Xu et al. [39]
PE	PA12	CB	PE	This study
PE	PA6	CB	Interface	This study

## Supplementary Materials

The following are available online at https://www.mdpi.com/1996-1944/13/11/2645/s1, Table S1: Electrical resistivity of filaments produced in this study by compounding in a twin-screw extruder, Table S2: Pilot melt-spinning: average spin pressures and processing temperatures, Table S3: Electrical resistivity of fibers produced in this study by melt-spinning, Table S4: Tensile properties of selected fibers produced in this study, Figure S1: Microscopic images of selected fiber cross-sections produced.
