# Supplementary material for: Antistatic Fibers for High-Visibility Workwear: Challenges of Melt-Spinning Industrial Fibers
Source: Materials (Basel). 2020 Jun 10;13(11):2645. doi: 10.3390/ma13112645 (PMC7321615; doi:10.3390/ma13112645)
Supplement: Supplementary file 1 [file materials-13-02645-s001.pdf]

materials

MDPI

Article

Antistatic Fibers for High-Visibility Workwear: Challenges of Melt-Spinning Industrial Fibers

Rudolf Hufenus [1,*], Ali Gooneie [1], Tutu Sebastian [2], Pietro Simonetti [1], Andreas Geiger [2], Dambarudhar Parida [1], Klaus Bender [3], Gunther Schäch [3] and Frank Clemens [2]

1 Laboratory for Advanced Fibers, Empa, Swiss Federal Laboratories for Materials Science and Technology, Lerchenfeldstrasse 5, 9014 St. Gallen, Switzerland; ali.gooneie@empa.ch (A.G.); pietro.simonetti@outlook.com (P.S.); dambarudhar.parida@empa.ch (D.P.)

2 Laboratory for High Performance Ceramics, Empa, Swiss Federal Laboratories for Materials Science and Technology, Überlandstrasse 129, 8600 Dübendorf, Switzerland; tutu.sebastian@empa.ch (T.S.); geigerandreas@web.de (A.G.); frank.clemens@empa.ch (F.C.)

3 EMS-CHEMIE AG, Business Unit EMS-GRILTECH, Via Innovativa 1, 7013 Domat/Ems, Switzerland; klaus.bender@emsgriltech.com (K.B.); gunther.schaech@emsgriltech.com (G.S.)

* Correspondence: rudolf.hufenus@empa.ch; Tel.: +41-58-765-7341

Supplementary Materials

Table S1. Electrical resistivity of filaments produced in this study by compounding in a twin-screw extruder (Minilab microcompounder if not stated otherwise). Specified are the compounds with respective CB and CNT content, as well as mean value and standard deviation of the measured electrical resistivity in Ωm.

Base Polymer		CB Compound		CNT Compound		CB Content (wt%)	CNT Content (wt%)	Electrical Resistivity
-	-	100%	6111	-	-	40	-	0.043 ± 0.000 Ωm
-	-	100%	6107	-	-	40	-	0.37 ± 0.01 Ωm
25%	5793	75%	6107	-	-	30	-	1.0 ± 0.1 Ωm
37.5%	5793	62.5%	6107	-	-	25	-	1.7 ± 0.2 Ωm
50%	5793	50%	6107	-	-	20	-	0.29 ± 0.01 kΩm
62.5%	5793	37.5%	6107	-	-	15	-	0.15 ± 0.02 MΩm
75%	5793	25%	6107	-	-	10	-	11 ± 5 MΩm
87.5%	5793	12.5%	6107	-	-	5	-	736 ± 151 MΩm
41.7%	5793	45%	6107	13.3%	6065	18	2	0.108 ± 0.003 kΩm
33.3%	5793	40%	6107	26.7%	6065	16	4	0.084 ± 0.002 kΩm
25%	5793	35%	6107	40%	6065	14	6	12.1 ± 0.6 Ωm
16.7%	5793	30%	6107	53.3%	6065	12	8	1.39 ± 0.08 Ωm
50%	HDPE	50%	6111	-	-	20	-	29.9 ± 1.4 MΩm
50%	MDPE	50%	6111	-	-	20	-	3.6 ± 0.4 MΩm
50%	LDPE	50%	6111	-	-	20	-	7.8 ± 0.4 MΩm
50%	5432	50%	6111	-	-	20	-	0.252 ± 0.000 Ωm
60%	5432	40%	6111	-	-	16	-	3.20 ± 0.09 Ωm
62.5%	5432	37.5%	6111	-	-	15	-	8.05 ± 0.13 Ωm
50%	5793	50%	6111	-	-	20	-	0.59 ± 0.01 Ωm
60%	5793	40%	6111	-	-	16	-	1.10 ± 0.13 Ωm
62.5%	5793	37.5%	6111	-	-	15	-	7.59 ± 0.70 Ωm
50%	5793	50%	6107	-	-	20	-	289 ± 5 Ωm
50%	5432	50%	6107	-	-	20	-	0.98 ± 0.04 MΩm
50%	LDPE	50%	6107	-	-	20	-	0.28 ± 0.32 MΩm
50%	887	50%	6111	-	-	20	-	0.23 ± 0.01 Ωm [1]

-	-	100%	6739	-	-	20	-	0.32 ± 0.15 kΩm [1]

[1] Compounded with twin screw extruder Collin.

Table S2. Pilot melt-spinning: average (±2) spin pressures and processing temperatures. The temperature of the polymer melt was measured between extruder and metering pump as well as between metering pump and spin pack.

**Antistatic compound**	**TiO_2 Content (wt%)**	**Spinneret Type**	**Anti-static Ratio (vol%)**	**Fibers No.**	**Spin Pressure (bar)**		**Polymer Melt Temperature (°C)**		
					minor part	major part	minor part	major part	spin pack
6107	0.9	wedge	20	1631-1635	85	145	255	276	281
			13	1636-1640	81	157	255	276	281
6066	0.9	sandwich	20	1581-1586	60	54	256	275	286
		wedge	20	1587-1591	64	53	256	275	281
			13	1592-1598	89	175	256	275	281
6082	0.9	wedge	20	1600-1604	93	123	257	277	281
			13	1605-1609	91	138	257	277	281
6081	0.9	sandwich	20	1654-1660	122	138	256	268	274
			13	1661-1667	112	145	256	270	274
		wedge	20	1610-1614	111	137	257	276	282
			13	1615-1619	107	151	257	276	282
6083	0.9	sandwich	20	1641-1646	195	140	256	260	259
			13	1647-1653	191	156	256	260	259
		wedge	20	1620-1624	185	166	256	270	285
			13	1625-1629	176	176	257	270	285
6237	0.9	sandwich	20	1669-1674	98	150	256	271	277
			13	1676-1682	92	162	256	271	279
	2.1	sandwich	20	1683-1688	95	149	256	272	278
			13	1690-1696	90	160	256	272	278

Table S3. Electrical resistivity of fibers produced in this study by melt-spinning. Specified are the bicomponent cross-section (sandwich or wedge), the type of antistatic compound (Table 1), the volumetric ratio of the antistatic compound in the bicomponent fiber, the respective draw ratio as well as mean value and standard deviation of the measured electrical resistivity in Ωm.

No.	Fiber Cross-Section	Antistatic Compound	Antistatic Ratio (vol%)	TiO2 Content (wt%)	Draw Ratio (DR)	Electrical Resistivity
1581	sandwich	6066	20	0.9	1.0	0.73 ± 0.09 Ωm
1583	sandwich	6066	20	0.9	2.0	3.4 ± 1.0 Ωm
1584	sandwich	6066	20	0.9	3.0	31 ± 7 kΩm
1585	sandwich	6066	20	0.9	1.5	0.71 ± 0.12 Ωm
1586	sandwich	6066	20	0.9	2.5	231 ± 7 Ωm
1587	wedge	6066	20	0.9	1.0	2.9 ± 0.4 Ωm
1588	wedge	6066	20	0.9	1.5	0.50 ± 0.11 Ωm
1589	wedge	6066	20	0.9	2.0	2.5 ± 1.1 Ωm
1590	wedge	6066	20	0.9	2.5	30 ± 16 Ωm
1591	wedge	6066	20	0.9	3.0	32 ± 4 kΩm
1592	wedge	6066	13	0.9	1.0	2.9 ± 2.3 Ωm
1593	wedge	6066	13	0.9	1.5	0.88 ± 0.11 Ωm
1594	wedge	6066	13	0.9	2.0	6.2 ± 0.5 Ωm
1595	wedge	6066	13	0.9	2.5	0.16 ± 0.06 kΩm
1596	wedge	6066	13	0.9	3.0	13 ± 3 kΩm
1597	wedge	6066	13	0.9	3.5	36 ± 14 kΩm
1598	wedge	6066	13	0.9	4.0	0.10 ± 0.14 MΩm
1600	wedge	6082	20	0.9	1.0	1.15 ± 0.07 Ωm
1601	wedge	6082	20	0.9	1.5	0.88 ± 0.07 Ωm
1602	wedge	6082	20	0.9	2.0	2.5 ± 0.4 Ωm
1603	wedge	6082	20	0.9	2.5	9.5 ± 0.7 Ωm
1604	wedge	6082	20	0.9	3.0	74 ± 5 Ωm
1605	wedge	6082	13	0.9	1.0	1.0 ± 0.2 Ωm
1606	wedge	6082	13	0.9	1.5	1.0 ± 0.1 Ωm
1607	wedge	6082	13	0.9	2.0	3.0 ± 0.4 Ωm
1608	wedge	6082	13	0.9	2.5	16 ± 3 Ωm
1609	wedge	6082	13	0.9	3.0	65.8 ± 0.5 Ωm
1610	wedge	6081	20	0.9	1.0	0.80 ± 0.22 Ωm
1611	wedge	6081	20	0.9	1.5	0.90 ± 0.07 Ωm
1612	wedge	6081	20	0.9	2.0	2.9 ± 0.1 Ωm
1613	wedge	6081	20	0.9	2.5	4.1 ± 0.3 Ωm
1614	wedge	6081	20	0.9	3.0	9.4 ± 0.7 Ωm
1615	wedge	6081	13	0.9	1.0	1.6 ± 0.4 Ωm
1616	wedge	6081	13	0.9	1.5	2.0 ± 0.1 Ωm
1617	wedge	6081	13	0.9	2.0	6.2 ± 1.2 Ωm
1618	wedge	6081	13	0.9	2.5	9.9 ± 3.6 Ωm
1619	wedge	6081	13	0.9	3.0	33 ± 5 Ωm
1620	wedge	6083	20	0.9	1.0	2.2 ± 0.3 Ωm
1621	wedge	6083	20	0.9	1.5	3.1 ± 0.4 Ωm
1622	wedge	6083	20	0.9	2.0	3.0 ± 0.3 Ωm
1623	wedge	6083	20	0.9	2.5	3.4 ± 0.2 Ωm
1624	wedge	6083	20	0.9	3.0	4.5 ± 1.6 Ωm
1625	wedge	6083	13	0.9	1.0	4.4 ± 0.6 Ωm
1626	wedge	6083	13	0.9	1.5	2.3 ± 0.2 Ωm
1627	wedge	6083	13	0.9	2.0	4.6 ± 0.4 Ωm

1628	wedge	6083	13	0.9	2.5	11 ± 1 Ωm
1629	wedge	6083	13	0.9	3.0	48 ± 4 Ωm
1631	wedge	6107	20	0.9	1.0	0.09 ± 0.02 Ωm
1632	wedge	6107	20	0.9	1.5	0.09 ± 0.01 Ωm
1633	wedge	6107	20	0.9	2.0	0.26 ± 0.04 Ωm
1634	wedge	6107	20	0.9	2.5	1.1 ± 0.2 Ωm
1635	wedge	6107	20	0.9	3.0	3.2 ± 0.2 Ωm
1636	wedge	6107	13	0.9	1.0	0.14 ± 0.02 Ωm
1637	wedge	6107	13	0.9	1.5	0.12 ± 0.04 Ωm
1638	wedge	6107	13	0.9	2.0	0.35 ± 0.03 Ωm
1639	wedge	6107	13	0.9	2.5	1.3 ± 0.3 Ωm
1640	wedge	6107	13	0.9	3.0	2.7 ± 0.6 Ωm
1641	sandwich	6083	20	0.9	1.0	3.4 ± 0.4 Ωm
1642	sandwich	6083	20	0.9	1.5	1.1 ± 0.1 Ωm
1643	sandwich	6083	20	0.9	2.0	3.7 ± 1.1 Ωm
1644	sandwich	6083	20	0.9	2.5	5.3 ± 0.6 Ωm
1645	sandwich	6083	20	0.9	3.0	61 ± 6 Ωm
1646	sandwich	6083	20	0.9	3.5	0.13 ± 0.02 kΩm
1647	sandwich	6083	13	0.9	1.0	3.7 ± 0.7 Ωm
1648	sandwich	6083	13	0.9	1.5	2.7 ± 0.6 Ωm
1649	sandwich	6083	13	0.9	2.0	5.5 ± 0.4 Ωm
1650	sandwich	6083	13	0.9	2.5	37 ± 3 Ωm
1651	sandwich	6083	13	0.9	3.0	0.11 ± 0.01 kΩm
1652	sandwich	6083	13	0.9	3.5	0.19 ± 0.02 kΩm
1653	sandwich	6083	13	0.9	4.0	1.1 ± 0.2 kΩm
1654	sandwich	6081	20	0.9	1.0	2.8 ± 0.6 Ωm
1655	sandwich	6081	20	0.9	1.5	1.2 ± 0.2 Ωm
1656	sandwich	6081	20	0.9	2.0	3.4 ± 0.2 Ωm
1657	sandwich	6081	20	0.9	2.5	12.4 ± 0.7 Ωm
1658	sandwich	6081	20	0.9	3.0	35 ± 1 Ωm
1659	sandwich	6081	20	0.9	3.5	63 ± 4 Ωm
1660	sandwich	6081	20	0.9	4.0	81 ± 9 Ωm
1661	sandwich	6081	13	0.9	1.0	3.1 ± 0.5 Ωm
1662	sandwich	6081	13	0.9	1.5	2.8 ± 0.1 Ωm
1663	sandwich	6081	13	0.9	2.0	7.9 ± 2.2 Ωm
1664	sandwich	6081	13	0.9	2.5	20 ± 2 Ωm
1665	sandwich	6081	13	0.9	3.0	62 ± 10 Ωm
1666	sandwich	6081	13	0.9	3.5	98 ± 11 Ωm
1667	sandwich	6081	13	0.9	4.0	0.19 ± 0.02 kΩm
1669	sandwich	6237	20	0.9	1.0	0.06 ± 0.04 kΩm
1670	sandwich	6237	20	0.9	1.5	37 ± 12 kΩm
1671	sandwich	6237	20	0.9	2.0	0.32 ± 0.01 MΩm
1672	sandwich	6237	20	0.9	2.5	0.25 ± 0.01 MΩm
1673	sandwich	6237	20	0.9	3.0	0.16 ± 0.01 MΩm
1674	sandwich	6237	20	0.9	3.5	0.15 ± 0.01 MΩm
1676	sandwich	6237	13	0.9	1.0	0.21 ± 0.05 kΩm
1677	sandwich	6237	13	0.9	1.5	7.4 ± 3.4 kΩm
1678	sandwich	6237	13	0.9	2.0	0.07 ± 0.02 MΩm
1679	sandwich	6237	13	0.9	2.5	0.19 ± 0.02 MΩm
1680	sandwich	6237	13	0.9	3.0	177 ± 4 kΩm
1681	sandwich	6237	13	0.9	3.5	172 ± 4 kΩm
1682	sandwich	6237	13	0.9	4.0	0.13 ± 0.03 MΩm

1683	sandwich	6237	20	2.1	1.0	0.05 ± 0.02 kΩm
1684	sandwich	6237	20	2.1	1.5	0.24 ± 0.01 MΩm
1685	sandwich	6237	20	2.1	2.0	78 ± 29 kΩm
1686	sandwich	6237	20	2.1	2.5	287 ± 4 kΩm
1687	sandwich	6237	20	2.1	3.0	0.24 ± 0.01 MΩm
1688	sandwich	6237	20	2.1	3.5	0.27 ± 0.01 MΩm
1690	sandwich	6237	13	2.1	1.0	0.10 ± 0.03 kΩm
1691	sandwich	6237	13	2.1	1.5	0.35 ± 0.07 MΩm
1692	sandwich	6237	13	2.1	2.0	0.42 ± 0.03 MΩm
1693	sandwich	6237	13	2.1	2.5	0.25 ± 0.01 MΩm
1694	sandwich	6237	13	2.1	3.0	0.28 ± 0.01 MΩm
1695	sandwich	6237	13	2.1	3.5	0.19 ± 0.03 MΩm
1696	sandwich	6237	13	2.1	4.0	0.17 ± 0.04 MΩm

Table S4. Tensile properties of selected fibers produced in this study. Specified are the bicomponent cross-section (sandwich or wedge), the volumetric ratio of the antistatic compound in the bicomponent fiber, the respective draw ratio and resulting fineness, as well as mean value and standard deviation of measured tensile properties. For comparison, the tensile properties of a monocomponent filament (No. 1563), melt-spun from PA6 Grilon A26, are also stated.

No.	Fiber Cross-Section	Antistatic Ratio (vol%)	Draw Ratio (DR)	Fineness (tex = mg/m)	Tensile Strength (cN/tex)	Strain at Break (%)
1563	mono	-	4.0	2.8	59.7 ± 1.4	52 ± 2
1620	wedge	20	1.0	12.7	12.0 ± 3.5	372 ± 28
1621	wedge	20	1.5	8.8	17.5 ± 2.7	223 ± 47
1622	wedge	20	2.0	6.3	21.2 ± 3.3	154 ± 13
1623	wedge	20	2.5	5.3	27.8 ± 3.4	99 ± 6
1624	wedge	20	3.0	4.5	44.8 ± 5.9	76 ± 5
1625	wedge	13	1.0	14.3	11.0 ± 1.1	374 ± 15
1626	wedge	13	1.5	9.5	16.9 ± 2.6	233 ± 21
1627	wedge	13	2.0	7.1	21.7 ± 1.9	153 ± 12
1628	wedge	13	2.5	5.8	27.1 ± 2.3	102 ± 7
1629	wedge	13	3.0	4.9	36.1 ± 2.9	73 ± 4
1641	sandwich	20	1.0	12.4	11.6 ± 4.6	290 ± 125
1642	sandwich	20	1.5	8.0	9.9 ± 1.5	195 ± 36
1643	sandwich	20	2.0	6.2	24.1 ± 8.4	172 ± 53
1644	sandwich	20	2.5	5.0	30.3 ± 3.9	105 ± 20
1645	sandwich	20	3.0	4.2	35.8 ± 3.8	75 ± 8
1646	sandwich	20	3.5	3.8	40.1 ± 15.7	46 ± 19
1647	sandwich	13	1.0	13.3	10.8 ± 1.4	368 ± 75
1648	sandwich	13	1.5	8.7	14.2 ± 1.2	228 ± 25
1649	sandwich	13	2.0	6.5	17.9 ± 2.4	134 ± 26
1650	sandwich	13	2.5	5.2	26.6 ± 3.6	105 ± 9
1651	sandwich	13	3.0	4.5	28.0 ± 2.3	69 ± 7
1652	sandwich	13	3.5	3.9	36.2 ± 2.9	54 ± 4
1653	sandwich	13	4.0	3.5	43.1 ± 2.1	39 ± 3

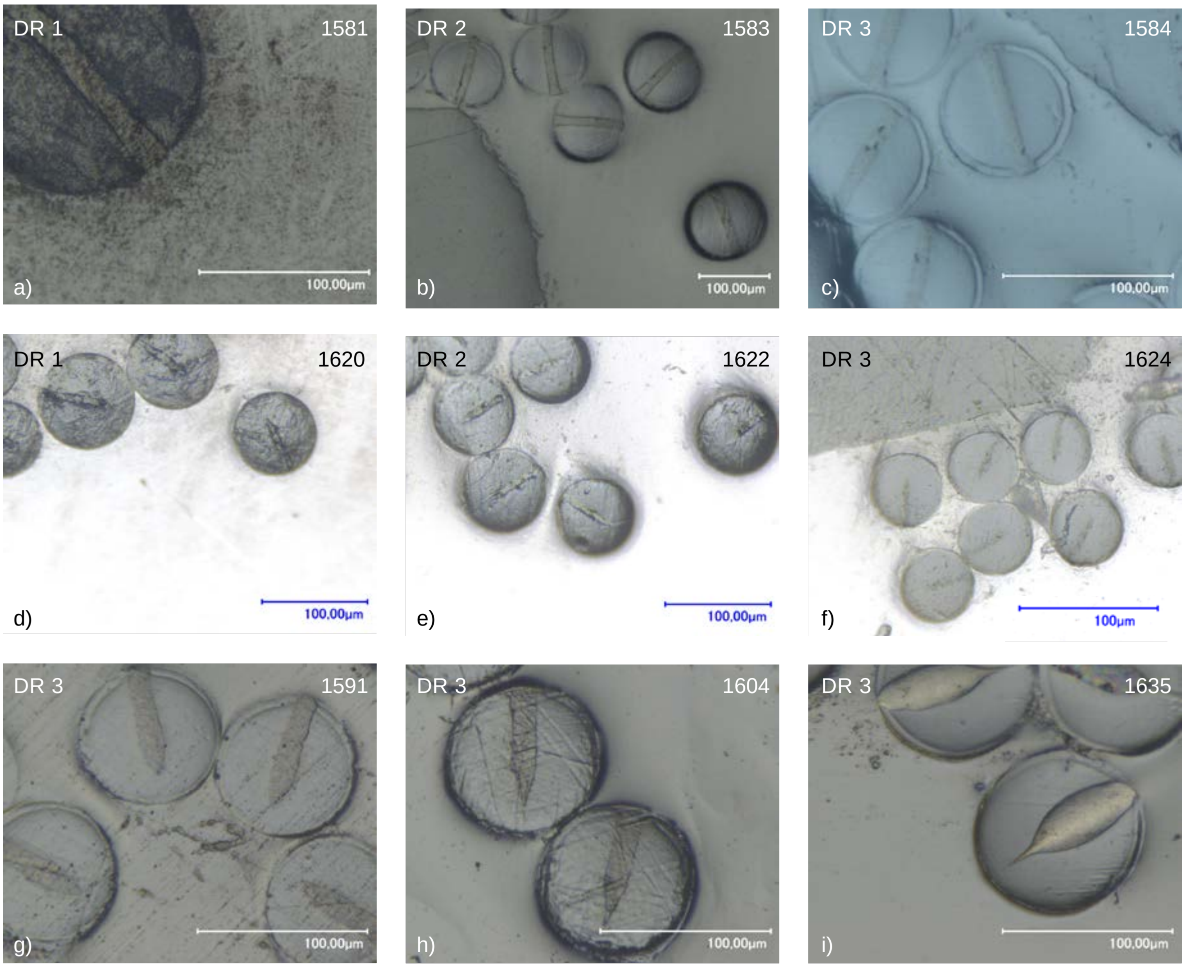

Figure S1. Microscopic images of selected fiber cross-sections produced: (**a**–**c**) sandwich fibers with draw ratios 1–3; (**d**–**f**) wedge fibers with draw ratios 1–3; (**g**–**i**) different types of wedge cross-sections (all draw ratio 3).
